# Epigenetic inheritance of cell fates during embryonic development

**DOI:** 10.3389/fgene.2014.00019

**Published:** 2014-02-04

**Authors:** Sirisha Cheedipudi, Oriana Genolet, Gergana Dobreva

**Affiliations:** ^1^Origin of Cardiac Cell Lineages Group, Max Planck Institute for Heart and Lung ResearchBad Nauheim, Germany; ^2^Medical Faculty, J. W. Goethe University FrankfurtFrankfurt, Germany

**Keywords:** epigenetic memory, epigenetics, cell identity, inheritance, cell fate, histone modification, ES cells

## Abstract

During embryonic development a large number of widely differing and specialized cell types with identical genomes are generated from a single totipotent zygote. Tissue specific transcription factors cooperate with epigenetic modifiers to establish cellular identity in differentiated cells and epigenetic regulatory mechanisms contribute to the maintenance of distinct chromatin states and cell-type specific gene expression patterns, a phenomenon referred to as epigenetic memory. This is accomplished via the stable maintenance of various epigenetic marks through successive rounds of cell division. Preservation of DNA methylation patterns is a well-established mechanism of epigenetic memory, but more recently it has become clear that many other epigenetic modifications can also be maintained following DNA replication and cell division. In this review, we present an overview of the current knowledge regarding the role of histone lysine methylation in the establishment and maintenance of stable epigenetic states.

## Introduction

Embryonic development involves the formation of highly complex tissues, which are comprised of many different cell types with specific and stable gene expression patterns. The role of tissue specific transcription factors is well established in regulating cell fate choices. A classical example is the master regulator of myoblast cell fate MyoD, the overexpression of which converts mouse embryonic fibroblasts into myoblasts (Davis et al., 1987). Similarly, overexpression of the key heart specific transcription factors GATA4, Mef2C, and Tbx5 reprograms embryonic and adult fibroblasts directly into cardiomyocytes (Ieda et al., 2010). Even more strikingly expression of Oct4, Sox2, c-Myc, and Klf4 converts fibroblasts into pluripotent stem cells (Takahashi and Yamanaka, 2006). Along with transcription factors, alterations in epigenetic modifications play an important role in cell fate decisions and differentiation during embryogenesis, where DNA replication provides a unique opportunity for a cell to change its epigenetic signature, thereby allowing pluripotent stem cells to differentiate in heterogeneous cell types. Later in development the cellular identity of distinct cell types has to be faithfully maintained through multiple rounds of cell divisions. The transmission of specific gene expression patterns through multiple rounds of cell divisions without changes of the DNA sequence and in the absence of instructive signals is referred to as epigenetic memory (Bird, 2002; Bernstein et al., 2007; Ptashne, 2007; Rivera and Ren, 2013). Epigenetic memory allows cells to maintain their identity, even when they are exposed to inductive signals guiding other cell fates (Bonasio et al., 2010; Blomen and Boonstra, 2011; Moazed, 2011). However, a certain level of plasticity must also be maintained, for example to enable restoration of tissue homeostasis following injury and other environmental challenges. A complex set of epigenetic modifications plays a key role in each of these situations, but our knowledge of how a cell switches from a state where epigenetic modifications are in flux to a state where they are stable—and back—remains limited. Historically, DNA methylation has been considered as the central mechanism responsible for epigenetic inheritance (Wigler, 1981; Sharif et al., 2007). However, more recent studies point to the inheritance of some post translational histone modifications through multiple rounds of cell division as an additional memory mechanism. In this review we present recent advances in understanding the role and the inheritance of epigenetic states with emphasis on histone lysine methylation (H3K4, H3K9, H3K27) during stem cell differentiation.

## Histone methylation in epigenetic inheritance of cell fates

Histone lysine methylation is considered an important player in epigenetic memory due to its relatively long half-life compared to other modifications (Barth and Imhof, 2010). Importantly, H3K4, H3K27, and H3K9 methylation have been shown to play critical function in epigenetic inheritance phenomena such as position effect variegation, Polycomb silencing and X chromosome inactivation (Rea et al., 2000; Bannister et al., 2001; Cao et al., 2002; Plath et al., 2004; Fodor et al., 2010).

### Bivalent domains

The presence of opposing epigenetic marks, termed “bivalency,” is thought to silence (H3K27me3) key lineage commitment genes while “poising” (H3K4me3) them for subsequent activation during differentiation, implying an important role for these modifications in cell fate decisions. Bivalent domains could resolve into active or repressive chromatin conformations depending on the abundance of the trithorax group (TrxG) or polycomb group (PcG) proteins during lineage commitment. Bivalent chromatin marks, as well as TrxG and PcG proteins are attractive candidates to serve as the epigenetic marks required for the maintenance of epigenetic memory, consistent with observations that they are stable following DNA replication (Alabert and Groth, 2012).

#### Trithorax and polycomb complexes in stem cell differentiation

The Trithorax complexes are evolutionarily conserved chromatin regulators, which activate transcription, and are divided into different classes based on their function (Schuettengruber et al., 2011). One class includes the SET domain-containing MLL histone methyltransferase complex which catalyzes the methylation of H3K4. Depletion of the integral core subunits of the MLL complex, Wdr5, Ash2l and RbBP5, results in global decrease of H3K4me3 levels, supporting a role of the MLL complex in establishing the H3K4me3 mark, a mark for transcriptionally active chromatin (Wysocka et al., 2005; Dou et al., 2006; Wan et al., 2013). Gene knockout studies of individual components of this complex highlighted their importance in embryonic development, ES cell pluripotency, lineage commitment, and differentiation (Supplemental Table 1). Deletion of either Mll1 or Mll2 leads to early embryonic lethality, suggesting that both Mll1 and Mll2 have important and non-redundant roles in development (Yu et al., 1995; Glaser et al., 2006). Mll2 deficiency affects ES cells proliferation, survival and differentiation as well as the timing and coordination of lineage commitment, but did not significantly affect pluripotency (Lubitz et al., 2007). In contrast, Wdr5 and Ash2l are essential for ES cells pluripotency (Ang et al., 2011; Wan et al., 2013). However, the role of the MLL complex in the regulation of the sequence of events that balance pluripotency vs. differentiation remain unknown.

The polycomb group (PcG) proteins act in complexes to silence genes via regulation of chromatin structure. In mammals, two major Polycomb group complexes exist: Polycomb repressive complex 1 (PRC1) and 2 (PRC2). PRC2 catalyzes the di- and trimethylation of H3 on Lys27, H3K27me2/3 (Cao et al., 2002; Czermin et al., 2002). The H3K27me2/3 mark is specifically recognized by the chromodomain of Polycomb (Pc), a subunit of PRC1 complexes, providing a platform for recruitment of the PRC1 complex (Wang et al., 2004). The PRC1 complex then ubiquitylates histone H2A on Lys119 (de Napoles et al., 2004; Fang et al., 2004) leading to Polycomb-mediated transcriptional repression. PRC1, however, can also be recruited in the absence of PRC2 and H3K27me3-enriched chromatin regions (Schoeftner et al., 2006). The core components of PRC2 are Suz12, Eed and Ezh1/2, which harbor the histone lysine methyltransferase activity in their SET domains. Ablation of all core PRC2 components is embryonically lethal due to severe defects at implantation and early post-implantation stages (Faust et al., 1995; O'Carroll et al., 2001; Pasini et al., 2004). Furthermore, depletion of Ezh1, Ezh2, Eed, and Suz12 in ES cells results in differentiation defects, reduced global H3K27me3 levels and de-repression of lineage specific genes (Boyer et al., 2006; Lee et al., 2006; Shen et al., 2008). Similarly, knockout of the key PRC1 core component Ring1B in ES cells leads to de-repression of PcG target genes and impairs differentiation (Leeb and Wutz, 2007; van der Stoop et al., 2008). Importantly, although loss of either PRC1 or PRC2 components results in aberrant gene expression and differentiation of ES cells, the combined loss of PRC1(Ring1b) and PRC2 (Ezh2) is not compatible with ES cell differentiation and survival, suggesting possible functional redundancy between the two complexes (Leeb et al., 2010).

Taken together these data firmly demonstrate that TrxG and PcG complexes are essential for proper embryonic development, ES cell pluripotency, lineage commitment, and differentiation because of their paramount role in the control of key developmental regulators.

#### Inheritance of bivalent domains

**Figure 1:**
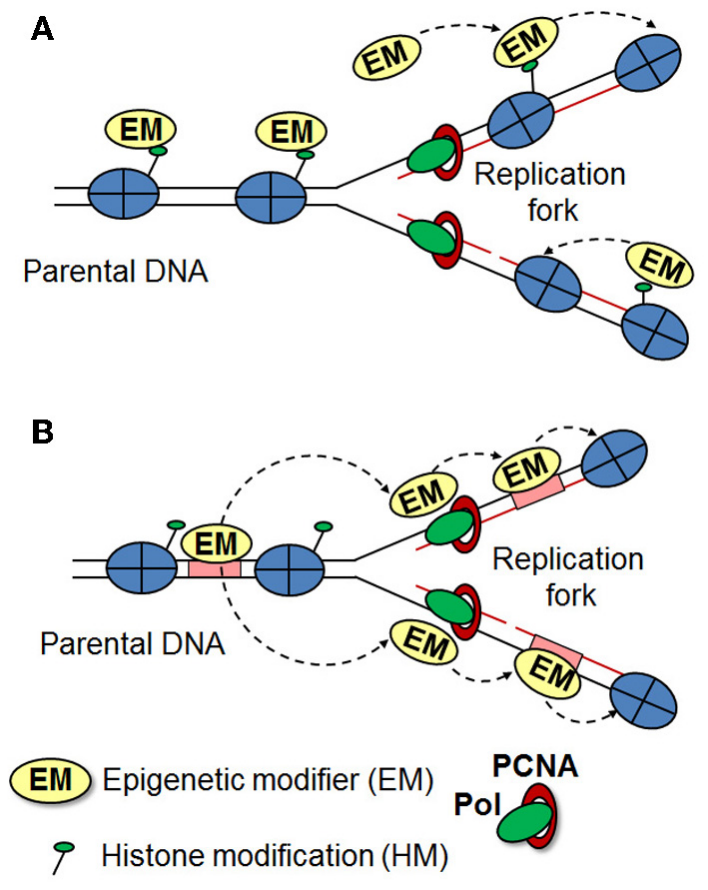
**Inheritance of histone modification patterns**. Two models for the maintenance of histone modifications are presented, which may apply in different organisms and cellular contexts. **(A)** Modified histones are evenly distributed between the daughter strands during replication, where they act as a template for further recruitment of histone modifiers to reestablish the original epigenetic state. **(B)** During replication, modified histones are rapidly replaced by unmethylated histones. Epigenetic modifiers (such as TrxG and PcG) remain stably associated with their binding elements (or other factors) during the progression of the replication fork and re-establish the histone modification patterns onto the newly deposited histones.

Epigenetic inheritance requires an “epigenetic mark,” which must be stable to DNA replication and should be recruited immediately to the newly synthesized DNA. However, when and how newly deposited histones acquire posttranslational modifications remains a matter of debate (Corpet and Almouzni, 2009; Zhu and Reinberg, 2011; Alabert and Groth, 2012). During DNA replication parental histones are distributed randomly between the two daughter DNA strands and it was suggested that histone modifying enzymes can recognize the posttranslational modifications on parental histones to help in reestablishing the specific modifications on newly deposited histones (Figure 1A) (Grewal and Moazed, 2003; Dodd et al., 2007; Kouzarides, 2007). In line with this, Hansen et al proposed that once a H3K27me3 mark is established, it recruits the PRC2 complex to maintain the mark at sites of DNA replication, even after the removal of the initiating signal (Hansen et al., 2008). However, a more recent study in *Drosophila* embryos showed that during S phase parental H3K4me3 and H3K27me3 are replaced by unmethylated histone H3 downstream of the DNA polymerase. Furthermore, using proximity ligation and re-ChIP assays, the authors demonstrated that Trithorax and Polycomb proteins remain associated to their response elements following the passage of the DNA polymerase to re-establish the histone modification patterns onto the newly deposited histones (Petruk et al., 2012). These data suggests that TrxG and PcG proteins, rather than H3K4me3 and H3K27me3, act as epigenetic marks required for memory (Figure 1B). However, whether this model is applicable for mammals remains to be determined. It is important to note, that while in *Drosophila* TrxG and PcG proteins are recruited to their response elements, in mammals there are very few examples of such sequences (Sing et al., 2009; Woo et al., 2010; Bengani et al., 2013). Hence, the mechanism responsible for their putative retention at sites of replication must be different. Interestingly, bivalent domains strongly correlate with CpG islands (Bernstein et al., 2006). Furthermore, artificial introduction of CpG islands leads to the establishment of H3K4me3 and H3K27me3 at these sites, pointing to a key role of CpG islands in the establishment and maintenance of bivalent domains (Mendenhall et al., 2010; Lynch et al., 2012). MLL1 and MLL2 proteins might target Trithorax complexes to bivalent promoters as these proteins possess zinc finger-CXXC domains, which specifically recognize unmethylated CpG islands (Birke et al., 2002). Similarly, the PRC1 component, KDM2B, which also harbors a CXXC domain, recruits PRC1 complexes to a subset of unmethylated CpG islands at bivalent promoters (Farcas et al., 2012). Furthermore, the histone variant H2A.Z might play a role in targeting and/or retention of MLL and PRC2 complexes at bivalent promoters, as knockdown of H2A.Z in ES cells leads to decreased occupancy of these complexes at bivalent promoters (Creyghton et al., 2008; Hu et al., 2013). Additional reports have highlighted the importance of transcription factors (Lee et al., 2006; Ang et al., 2011) and non-coding RNAs in targeting trithorax and polycomb complexes (Marchese and Huarte, 2013; Fatica and Bozzoni, 2014). Although these studies contribute to our understanding of the establishment of H3K4me3 and H3K27me3 marks at bivalent domains in ES cell based systems, much remains to be learned about the establishment and maintenance of these domains at specific genetic loci in the various specialized cell types during development and in adults.

### H3K9 methylation

H3K9me is a major epigenetic hallmark of heterochromatin, which is stably inherited during cell division. Heterochromatin is critical for genomic stability, centromere function, silencing of repetitive DNA elements as well as gene regulation and cell fate determination. In mammalian cells H3K9 methylation is catalyzed by Setdb1 (Eset), G9a, Suv39h1, Suv39h2, and Ehmt1 (Eu-HMTase). Suv39h proteins are preferentially targeted to the pericentric heterochromatin, and mice lacking both Suv39h1 and Suv39h2 show chromosomal instabilities and increased risk of cancer, associated with H3K9me loss (Peters et al., 2001). By contrast, G9a plays a key role in embryonic development. Ablation of G9a leads to dramatic loss of DNA methylation, HP1 binding to euchromatin and embryonic lethality at E8.5-E9.5 (Tachibana et al., 2002, 2008). Furthermore, G9a is required for cell fate determination through silencing of the key pluripotency gene Oct3/4 (Feldman et al., 2006). Mutation of Setdb1 in mice leads to peri-implantation lethality and Setdb1 knockout ES cells cannot be established (Dodge et al., 2004), presumably due to the key role of Setdb1 in restricting the lineage commitment of ES cells toward extraembryonic cell fates by interaction with Oct4 at trophoblast associated genes (Yeap et al., 2009; Yuan et al., 2009). Together, these data support a key role of H3K9 methylation in embryonic development and lineage commitment.

**Figure 2:**
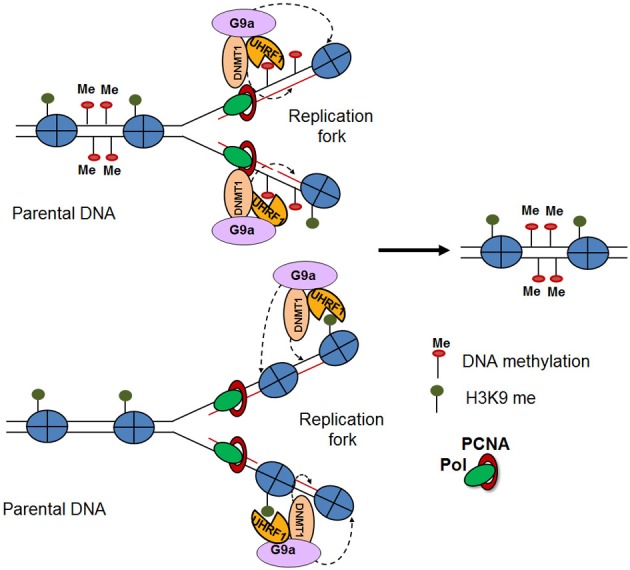
**Modification reinforcement model (Zhu and Reinberg, 2011)**. DNA replication results in hemi-methylated daughter DNA strands. UHRF1 recognizes the hemi-methylated DNA and recruits DNMT1 to restore the DNA methylation pattern on the newly-synthesized DNA. The interaction of DNMT1 with G9a, results in posttranslational modifications of the newly deposited histones (top). Furthermore, H3K9me2/3 mark recruits UHRF1 and DNMT1-G9a, thereby facilitating DNA and H3K9 methylation (bottom).

Several studies have provided mechanistic insights in the inheritance of H3K9me3 methylation and heterochromatic states. Interestingly, heterochromatic regions feature high density methylation of CpG islands along with H3K9me3 methylation (Fahrner et al., 2002; Lehnertz et al., 2003). Indeed, it was demonstrated that the methyl CpG binding domain 1 (MBD1) protein recruits Setdb1 to the chromatin accessibility factor (CAF1) during S phase, facilitating the methylation of newly deposited histone H3 at K9 (Sarraf and Stancheva, 2004). Similarly, the DNA methyltransferase, DNMT1, which binds to hemi-methylated daughter DNA strands during replication, directly interacts with G9a at the replication fork. Both proteins are loaded as a complex onto the chromatin along with PCNA, resulting in H3K9me3 of the newly deposited histones (Esteve et al., 2005). On the other hand, H3K9me2/3 can recruit UHRF1, a factor involved in the loading of DNA methyltransferases, thereby facilitating DNA methylation (Karagianni et al., 2008). The cross talk and mutually reinforcing nature of these different epigenetic mechanisms appear to ensure long-term cellular memory (Figure 2) (Zhu and Reinberg, 2011). Together, these studies suggest an important connection between DNA methylation and the stable inheritance of heterochromatic states (Sarraf and Stancheva, 2004). However, a recent study demonstrated that the maintenance of induced heterochromatin is not dependent on DNA methylation. Using a chromatin *in vivo* assay (CiA), which enables induction and termination of chromatin modifications in living cells, the authors selectively targeted HP1α to induce a H3K9me3 heterochromatic domain at the Oct4 locus (Hathaway et al., 2012). Interestingly, they found that after removal of HP1α these heterochromatic domains were heritably transmitted over multiple cell divisions independently of DNA methylation, suggesting that H3K9me3 is the epigenetic mark required for inheritance of heterochromatic state. This highlights the complexity of the mechanisms by which H3K9me3 is maintained through cell division and the need for their further investigation.

### Histone demethylases in epigenetic inheritance of cell fates

Histone lysine methylation is regulated dynamically by methylases and demethylases. Lsd1 is the first demethylase that was discovered. Interestingly, the demethylase specificity and activity of Lsd1 appear to be determined by its binding partners. Lsd1 acts as an H3K4me2/me1 demethylase in association with the CoREST repressor complex, and as H3K9me2/me1 demethylase in a complex with the androgen receptor (Shi et al., 2004; Metzger et al., 2005). Ablation of Lsd1 leads to early embryonic lethality and Lsd1-deficient ES cells show defective differentiation and increased cell death associated with progressive loss of DNA methylation (Wang et al., 2009). The loss of DNA methylation was due to the role of Lsd1 in regulating the stability of Dnmt1, by direct demethylation (Wang et al., 2009). However, it was recently shown that Lsd1 plays a more direct role in the regulation of the epigenetic states of critical developmental regulators, by regulating the balance between H3K4 and H3K27 methylation at their regulatory regions (Adamo et al., 2011). The H3K4me3 demethylase, Jarid1a, and the H3K27me3 demethylases, Jmjd3 and UTX, counteract the TrxG and PcG complexes, thereby helping to resolve the bivalent domains during ES cell differentiation. Jarid1a is recruited by the PRC2 complex to PcG target genes in ES cells to repress their expression (Pasini et al., 2008). During ES cell differentiation Jarid1a dissociates from the classical PcG target genes, the Hox genes, resulting in an increased H3K4me3 levels and gene activation (Christensen et al., 2007). However, Jarid1a knockout mice are viable and display mild phenotype, which is probably due to redundancy with other Jarid1 family members (Klose et al., 2007). The H3K27me3 demethylase, UTX, associates with the MLL complex and the UTX/MLL complex is recruited to the Hox gene cluster upon retinoic acid signaling. This leads to demethylation of H3K27me3 and concomitant increases in H3K4me3 leading to transcriptional activation (Agger et al., 2007; Lee et al., 2007). The H3K27me3 demethylase, Jmjd3 regulates the expression of Brachyury, a key player in mesoderm formation, by recruiting β-catenin to the Brachyury promoter (Ohtani et al., 2013). Consistent with this, ablation of Jmjd3 is early embryonic lethal and Jmjd3-deficient ES cells showed compromised mesodermal differentiation (Ohtani et al., 2013). The histone H3K9 demethylases, Jmjd1a and Jmjd2c, are required for ES cell pluripotency by binding to and positively regulating the pluripotency-associated genes Nanog, Tcl1, Tcfcp2l1, and Zfp57 (Loh et al., 2007). Although these studies demonstrate that histone demethylases are essential for proper embryonic development, ES cell pluripotency and lineage commitment and contribute to our understanding of the dynamic regulation of H3K4, H3K9, and H3K27 methylation marks, future studies are needed to understand the interplay between histone methylases and demethylases in the balance and transition between dynamic and stable epigenetic states.

## Perspectives

The recent years have brought major advances in the understanding of the epigenetic marks that form the basis of epigenetic memory and the general mechanisms through which they can be replicated and inherited. Additionally, studies in ES cells and knockout mice have revealed key functions of epigenetic modifiers in the establishment of epigenetic states, regulating ES cell pluripotency and differentiation. While central molecular players responsible for the establishment and stable maintenance of various epigenetic modifications have been identified, much remains to be learned about the variations and fine-tuning of these general mechanisms at specific genetic loci or larger chromosomal domains, as well as within distinct cellular contexts. A myriad of studies using conditional knockout approaches have provided evidence of important functions of epigenetic modifiers in organ formation. However, most of this work focused on phenotypic characterization and analysis of gene expression patterns, whereas the detailed molecular mechanism of their function in the establishment and maintenance of stable epigenetic states, resulting in stable cell identities remain poorly understood. One major drawback of most studies so far has been the use of whole organs and ES/EB differentiation systems to analyze the role of epigenetic modifiers in cell fate determination and differentiation during development. Organs and EBs consist of large number of heterogeneous cell types with distinct cell-type expression patterns, which makes it difficult to understand the chromatin events occurring in cell fate choices and differentiation in particular lineages. Chromatin analysis (ChIP-seq, MNase-seq, DNA methylation analysis, etc.) combined with state-of-the-art imaging technologies and interaction analyses in homogeneous cell populations of sorted stem/progenitor cells and their lineage-traced progeny would contribute to better understanding of the epigenetic marks required for the establishment and maintenance of cellular identity. Additionally, it would be important to address whether and how transcriptional master regulators of cell fate work together with epigenetic modifiers in the establishment of cell-type specific gene expression patterns during cell fate determination and differentiation. Another fundamental issue is that while epigenetic memory plays an important role in maintaining cellular identity through conservation of specific epigenetic modifications, the same epigenetic modifications must be flexible and variable, e.g., during development, stress or regeneration. Future studies are needed to analyze the role of histone methyltransferases and demethylases in regulating cell identity and cell plasticity. Clarifying the mechanisms regulating the balance and transition between dynamic and stable epigenetic states will likely constitute a major area for future study in this field.

### Conflict of interest statement

The authors declare that the research was conducted in the absence of any commercial or financial relationships that could be construed as a potential conflict of interest.

## Supplementary material

Supplemental Table 1 **Function of histone methyltransferases and demethylases involved in epigenetic inheritance during development and ES cell differentiation**.
