# Supplementary material for: Epigenetic inheritance of cell fates during embryonic development
Source: Front Genet. 2014 Feb 4;5:19. doi: 10.3389/fgene.2014.00019 (PMC3912789; doi:10.3389/fgene.2014.00019)
Supplement: Supplemental Table 1 — Function of histone methyltransferases and demethylases involved in epigenetic inheritance during development and ES cell differentiation. [file DataSheet1.DOCX]

**Supplemental Table 1.** Function of histone methyltransferases and demethylases involved in epigenetic inheritance during development and ES cell differentiation.

| **Protein or complex** | **Function** | **Subunits/**  **Gene (mouse)** | **Knockout mouse phenotype** | **Role in ES cell pluripotency and differentiation** | **References** |
| --- | --- | --- | --- | --- | --- |
| **Histone H3 lysine 4 methylation** | | | | | |
| **Trx** | Methylates H3K4, transcriptional activation, regulation of Hox genes, regulation of cell identity | Mll1 | Embryonic lethal at E12.5, impaired Hox gene expression, segmental identity defects, reduction in hematopoietic progenitors, defects in fetal liver and postnatal hematopoiesis. | Embryoid bodies fail to differentiate towards hematopoietic progenitors. | ([Yu et al., 1995](#_ENREF_77);[Yagi et al., 1998](#_ENREF_74);[Ernst et al., 2004](#_ENREF_23);[Terranova et al., 2006](#_ENREF_63);[Gan et al., 2010](#_ENREF_26)) |
|  |  | Mll2 | Embryos die before E11.5, show increased apoptosis, developmental delay and loss of Hox genes expression. Deletion after E11.5 results in male sterility.  Conditional deletion in oocytes leads to global reduction in H3K4me3 levels, death of oocytes. | Pluripotency is unaffected, however, defective proliferation, increased apoptosis, compromised coordination and timing of early differentiation was observed. | ([Glaser et al., 2006](#_ENREF_28);[Lubitz et al., 2007](#_ENREF_41);[Glaser et al., 2009](#_ENREF_27);[Andreu-Vieyra et al., 2010](#_ENREF_5);[Hu et al., 2013](#_ENREF_29)) |
|  |  | Wdr5 |  | ES cell pluripotency is affected. Interacts with Oct4 and regulates expression of pluripotency genes. Wdr5-deficiency leads to global reduction in H3K4me3 levels. | ([Wysocka et al., 2005](#_ENREF_73);[Ang et al., 2011](#_ENREF_6)) |
|  |  | Ash2l | Embryonic lethal before E8.5. | Ash2l-/- ES cells cannot be derived. Depletion leads to reduction in H3K4me3 levels. Regulates pluripotency by maintaining open chromatin configuration. | ([Stoller et al., 2010](#_ENREF_58);[Wan et al., 2013](#_ENREF_68)) |
|  |  | Rbbp5 |  | Differentiation of Rbbp5-depleted ES cells is affected, due to failure in induction of key developmental genes. | ([Jiang et al., 2011](#_ENREF_31)) |
| **H3K4me1-3 demethylases** | H3K4me2/1 demethylase  H3K9me2/me1 demethylase | Lsd1 | Embryonic lethal before E6.5. | Defective differentiation, increased cell death. Regulates pluripotency by occupying promoters of developmental regulatory genes with bivalent domains. Regulates stem cell proliferation. | ([Wang et al., 2007](#_ENREF_70);[Wang et al., 2009](#_ENREF_71);[Adamo et al., 2011](#_ENREF_1);[Yin et al., 2014](#_ENREF_76)) |
|  | H3K4me2/1 demethylase | Lsd2 | Mice develop normally. Loss of DNA methylation on imprinted genes; embryos derived from null oocytes show biallelic expression or silencing of imprinted genes. |  | ([Ciccone et al., 2009](#_ENREF_15)) |
|  | H3K4me3/2  demethylase | Jarid1a | Mice are viable and have normal life span. | PRC2 complex recruits Jarid1a to repress developmental regulators. Dissociate from Hox genes during ES cell differentiation. | ([Christensen et al., 2007](#_ENREF_14);[Pasini et al., 2008](#_ENREF_50);[Lin et al., 2011](#_ENREF_38)) |
|  | H3K4me3/2  demethylase | Jarid1b | Neonatal lethality due to respiratory failure. Embryos display defects in neural and eye development, homeotic skeletal transformations, increased expression of neural master regulatory genes. Aberrant accumulation of H3K4me3 during early embryogenesis. | ES cell pluripotency is unaffected. Differentiation towards neural lineage is compromised. Depletion leads to global increase in H3K4me3. Occupies promoters of developmental regulators. Plays a role in expansion of proliferating progenitor cells. | ([Dey et al., 2008](#_ENREF_19);[Catchpole et al., 2011](#_ENREF_12);[Schmitz et al., 2011](#_ENREF_53);[Albert et al., 2013](#_ENREF_4)) |
| **Histone H3 lysine 27 methylation** | | | | | |
| **PRC2** | Methylates H3K27, transcriptional repression, Hox genes repression, regulates cell fate decisions | Ezh2 | Complete knockout is early post implantation lethal (around E7.5-E8.5). Conditional deletion of maternal Ezh2 affects the localization of the Eed protein and leads to epigenetic asymmetry of H3K9me3 and H3K27me3 in the parental genomes. | Mesoendodermal differentiation of ES cells is compromised. Deficiency leads to decrease in H3K27me2 and H3K27me3, however, H3K27me3 is significantly retained at promoters of developmental regulators due to partial redundancy with Ezh1. Regulates the epigenetic state of the Nanog promoter, thereby regulating the equilibrium between self-renewal and differentiation of ES cells. | ([O'Carroll et al., 2001](#_ENREF_45);[Erhardt et al., 2003](#_ENREF_22);[Shen et al., 2008](#_ENREF_55);[Villasante et al., 2011](#_ENREF_66)) |
|  |  | Ezh1 |  | Complements Ezh2 partially, depletion of Ezh1 in Ezh2-/- ES cells abolishes residual H3K27me3 levels. | ([Shen et al., 2008](#_ENREF_55)) |
|  |  | Eed | Defects in the mesoderm formation, die around E8.5. Defects in anterior-posterior patterning. | ES cell pluripotency is unaffected. Differentiation is affected due to derepression of key developmental regulators. Eed deficiency leads to global reduction in H3K27me1/2/3 levels and reduced Ezh2 protein levels. | ([Faust et al., 1995](#_ENREF_24);[Schumacher et al., 1996](#_ENREF_54);[Montgomery et al., 2005](#_ENREF_42);[Boyer et al., 2006](#_ENREF_9);[Chamberlain et al., 2008](#_ENREF_13);[Leeb et al., 2010](#_ENREF_36)) |
|  |  | Suz12 | Embryonic lethal at E7.5-E8.5, Suz12-deficiency leads to proliferative defects and loss of H3K27me2 and H3K27me3 | Improper differentiation, global loss of H3K27me2 and H3K27me3, higher expression of differentiation specific genes, decrease in Ezh2 protein levels. | ([Pasini et al., 2004](#_ENREF_49);[Pasini et al., 2007](#_ENREF_48)) |
| **H3K27me2/3 demethylases** | H3K27me2/3 demethylase | Utx | Female embryos are more affected than males, show reduced number of somites, defects in neural tube closure, heart malformations, female embryos die around E12.5, some male embryos survive to adulthood. | ES cell pluripotency is unaffected. Defects in differentiation towards mesoderm and ectoderm. Utx depletion leads to an increase of H3K27me3 at transcriptional start sites of developmental regulatory genes. | ([Shpargel et al., 2012](#_ENREF_56);[Wang et al., 2012](#_ENREF_69);[Welstead et al., 2012](#_ENREF_72);[Morales Torres et al., 2013](#_ENREF_43)) |
|  | H3K27me2/3 demethylase | Jmjd3 | Embryonic lethal before E6.5. | Pluripotency of ES cells is unaffected. Compromised mesodermal and endodermal differentiation. Mild increase in global H3K27me3 levels. | ([Burgold et al., 2008](#_ENREF_10);[Burgold et al., 2012](#_ENREF_11);[Ohtani et al., 2013](#_ENREF_47)) |
| **Histone H2A ubiquitination (H2AK119ub)** | | | | | |
| **PRC1** | Ubiquitination of H2A on lysine 119, chromatin compaction, transcriptional repression, regulates cell fate decisions | Ring1a | Both homozygous and heterozygous null mice show anterior transformations and abnormalities of axial skeleton. | Ring1a/1b double knock-out ES cells display defects in proliferation, loss of H2Aub, release of poised RNAP and derepression of developmental regulators. | ([del Mar Lorente et al., 2000](#_ENREF_18);[Stock et al., 2007](#_ENREF_57);[Endoh et al., 2008](#_ENREF_21)) |
|  |  | Ring1b | Embryonic lethal around E10.5. Defects of embryonic and extraembryonic tissues during gastrulation. A hypomorphic allele exhibit posterior homeotic transformation of the axial skeleton and mild derepression of some Hox genes. | ES cells retain expression of key pluripotency regulators, show abnormal differentiation and derepression of lineage genes. Leads to loss of H2Aub and reduction in the protein levels of members of the PRC1 complex. | ([Suzuki et al., 2002](#_ENREF_59);[Voncken et al., 2003](#_ENREF_67);[de Napoles et al., 2004](#_ENREF_17);[Leeb and Wutz, 2007](#_ENREF_37);[van der Stoop et al., 2008](#_ENREF_65)) |
|  |  | Bmi1 | Mice die between 3-20 weeks after birth, display posterior homeotic transformation, neurological abnormalities and hematopoietic defects. | Depletion by RNAi does not affect pluripotency but affects differentiation. Impaired expression of the primitive endodermal markers Gata6 and Sox17. | ([van der Lugt et al., 1994](#_ENREF_64);[Lavial et al., 2012](#_ENREF_35)) |
|  |  | Mel18 | Die around 4 weeks after birth. Display growth retardation and posterior transformations of axial skeleton.  Bmi1-/-Mel18-/- double knockout is embryonically lethal. |  | ([Akasaka et al., 1996](#_ENREF_2);[Akasaka et al., 2001](#_ENREF_3)) |
|  |  | Phc1,2 | Phc1-/- mice show perinatal lethality, posterior skeletal transformation and neural crest defects.  Phc2-/- mice are viable, display homeotic transformation of the axial skeleton and derepression of Hox genes.  Phc1-/-Phc2-/- double null mice die at E9.5. |  | ([Takihara et al., 1997](#_ENREF_62);[Isono et al., 2005](#_ENREF_30)) |
|  |  | Cbx2,4,6,7,8 | Cbx2-/- mice show postnatal lethality from birth to 4-6 weeks of age, growth retardation, homeotic transformation of the skeleton and male to female sex reversal.  Cbx7-/- mice are viable, develop liver and lung adenocarcinomas. | Cbx2, as well as Cbx4 depletion in ES cells by RNAi leads to aberrant expression of mesoderm and endoderm markers.  Cbx7 depletion leads to aberrant ES cell differentiation and deregulation of ectodermal and mesodermal lineage genes. | ([Core et al., 1997](#_ENREF_16);[Katoh-Fukui et al., 1998](#_ENREF_32);[Forzati et al., 2012](#_ENREF_25);[Morey et al., 2012](#_ENREF_44);[O'Loghlen et al., 2012](#_ENREF_46)) |
| **Histone H3 lysine 9 methylation** | | | | | |
| **H3K9me1-3 methyl transferases** | Establish H3K9me3 at constitutive pericentromeric and telomeric heterochromatic regions | Suv39h1,  Suv39h2 | Mice deficient for either Suv39h1 or Suv39h2 are viable and fertile. Some double null mice are viable. More severe phenotypes include growth retardation, chromosomal instabilities with increased risk of tumors. Leads to a loss of H3K9me3 from heterochromatin. |  | ([Peters et al., 2001](#_ENREF_52)) |
|  | H3K9 methylation mainly of euchromatic regions, transcriptional repression | Setdb1  (Eset) | Peri-implantation lethality between E3.5-E5.5 | ES cells cannot be derived. Knockdown results in loss of ES cell morphology, up regulation of a set of bivalently marked developmental regulators and differentiation towards the trophectodermal lineage. Global H3K9me2 and me3 levels are unchanged. | ([Dodge et al., 2004](#_ENREF_20);[Bilodeau et al., 2009](#_ENREF_8);[Yeap et al., 2009](#_ENREF_75);[Yuan et al., 2009](#_ENREF_78)) |
|  |  | Glp (Ehmt1) | Embryonic lethality around E9.5, reduction of H3K9me2. | Reduction in H3K9me1 and me2, H3K9me3 levels unchanged. | ([Tachibana et al., 2002](#_ENREF_60);[Tachibana et al., 2005](#_ENREF_61)) |
|  |  | G9a (Ehmt2) | Embryos die around E9.5-E12.5, growth retardation, global loss of H3K9me2. | ES cell pluripotency is unaffected. Growth and survival are affected during ES cell differentiation. | ([Tachibana et al., 2002](#_ENREF_60);[Tachibana et al., 2005](#_ENREF_61)) |
| **H3K9me1-3 demethylases** | H3K9me2/me1 demethylase | Jmjd1a | Male to female sex reversal, abnormal spermatogenesis, obesity associated hyperlipidemia. Increase in H3K9me1and H3K9me2 levels. | Depletion leads to loss of pluripotency and increased expression of lineage commitment genes. | ([Loh et al., 2007](#_ENREF_40);[Liu et al., 2010](#_ENREF_39);[Kuroki et al., 2013](#_ENREF_34)) |
|  | H3K9me3/me2 demethylase | Jmjd2b | No overt phenotype. | Knock down affects ES cell self-renewal. Conditional deletion leads to reduction of H3K9me3 levels. | ([Kawazu et al., 2011](#_ENREF_33);[Antony et al., 2013](#_ENREF_7)) |
|  | H3K9me3/me2 demethylase | Jmjd2c | Not required for embryonic development. | Knockdown of Jmjd2c leads to loss of pluripotency and induced differentiation, however, Jmjd2c -/- ES cells maintained undifferentiated morphology and displayed a normal growth rate. | ([Loh et al., 2007](#_ENREF_40);[Pedersen et al., 2014](#_ENREF_51)) |

**Supplemental References:**

Adamo, A., Sese, B., Boue, S., Castano, J., Paramonov, I., Barrero, M.J., and Izpisua Belmonte, J.C. (2011). LSD1 regulates the balance between self-renewal and differentiation in human embryonic stem cells. *Nat Cell Biol* 13**,** 652-659.

Akasaka, T., Kanno, M., Balling, R., Mieza, M.A., Taniguchi, M., and Koseki, H. (1996). A role for mel-18, a Polycomb group-related vertebrate gene, during theanteroposterior specification of the axial skeleton. *Development* 122**,** 1513-1522.

Akasaka, T., Van Lohuizen, M., Van Der Lugt, N., Mizutani-Koseki, Y., Kanno, M., Taniguchi, M., Vidal, M., Alkema, M., Berns, A., and Koseki, H. (2001). Mice doubly deficient for the Polycomb Group genes Mel18 and Bmi1 reveal synergy and requirement for maintenance but not initiation of Hox gene expression. *Development* 128**,** 1587-1597.

Albert, M., Schmitz, S.U., Kooistra, S.M., Malatesta, M., Morales Torres, C., Rekling, J.C., Johansen, J.V., Abarrategui, I., and Helin, K. (2013). The histone demethylase Jarid1b ensures faithful mouse development by protecting developmental genes from aberrant H3K4me3. *PLoS Genet* 9**,** e1003461.

Andreu-Vieyra, C.V., Chen, R., Agno, J.E., Glaser, S., Anastassiadis, K., Stewart, A.F., and Matzuk, M.M. (2010). MLL2 is required in oocytes for bulk histone 3 lysine 4 trimethylation and transcriptional silencing. *PLoS Biol* 8.

Ang, Y.S., Tsai, S.Y., Lee, D.F., Monk, J., Su, J., Ratnakumar, K., Ding, J., Ge, Y., Darr, H., Chang, B., Wang, J., Rendl, M., Bernstein, E., Schaniel, C., and Lemischka, I.R. (2011). Wdr5 mediates self-renewal and reprogramming via the embryonic stem cell core transcriptional network. *Cell* 145**,** 183-197.

Antony, J., Oback, F., Chamley, L.W., Oback, B., and Laible, G. (2013). Transient JMJD2B-mediated reduction of H3K9me3 levels improves reprogramming of embryonic stem cells into cloned embryos. *Mol Cell Biol* 33**,** 974-983.

Bilodeau, S., Kagey, M.H., Frampton, G.M., Rahl, P.B., and Young, R.A. (2009). SetDB1 contributes to repression of genes encoding developmental regulators and maintenance of ES cell state. *Genes Dev* 23**,** 2484-2489.

Boyer, L.A., Plath, K., Zeitlinger, J., Brambrink, T., Medeiros, L.A., Lee, T.I., Levine, S.S., Wernig, M., Tajonar, A., Ray, M.K., Bell, G.W., Otte, A.P., Vidal, M., Gifford, D.K., Young, R.A., and Jaenisch, R. (2006). Polycomb complexes repress developmental regulators in murine embryonic stem cells. *Nature* 441**,** 349-353.

Burgold, T., Spreafico, F., De Santa, F., Totaro, M.G., Prosperini, E., Natoli, G., and Testa, G. (2008). The histone H3 lysine 27-specific demethylase Jmjd3 is required for neural commitment. *PLoS One* 3**,** e3034.

Burgold, T., Voituron, N., Caganova, M., Tripathi, P.P., Menuet, C., Tusi, B.K., Spreafico, F., Bevengut, M., Gestreau, C., Buontempo, S., Simeone, A., Kruidenier, L., Natoli, G., Casola, S., Hilaire, G., and Testa, G. (2012). The H3K27 demethylase JMJD3 is required for maintenance of the embryonic respiratory neuronal network, neonatal breathing, and survival. *Cell Rep* 2**,** 1244-1258.

Catchpole, S., Spencer-Dene, B., Hall, D., Santangelo, S., Rosewell, I., Guenatri, M., Beatson, R., Scibetta, A.G., Burchell, J.M., and Taylor-Papadimitriou, J. (2011). PLU-1/JARID1B/KDM5B is required for embryonic survival and contributes to cell proliferation in the mammary gland and in ER+ breast cancer cells. *Int J Oncol* 38**,** 1267-1277.

Chamberlain, S.J., Yee, D., and Magnuson, T. (2008). Polycomb repressive complex 2 is dispensable for maintenance of embryonic stem cell pluripotency. *Stem Cells* 26**,** 1496-1505.

Christensen, J., Agger, K., Cloos, P.A., Pasini, D., Rose, S., Sennels, L., Rappsilber, J., Hansen, K.H., Salcini, A.E., and Helin, K. (2007). RBP2 belongs to a family of demethylases, specific for tri-and dimethylated lysine 4 on histone 3. *Cell* 128**,** 1063-1076.

Ciccone, D.N., Su, H., Hevi, S., Gay, F., Lei, H., Bajko, J., Xu, G., Li, E., and Chen, T. (2009). KDM1B is a histone H3K4 demethylase required to establish maternal genomic imprints. *Nature* 461**,** 415-418.

Core, N., Bel, S., Gaunt, S.J., Aurrand-Lions, M., Pearce, J., Fisher, A., and Djabali, M. (1997). Altered cellular proliferation and mesoderm patterning in Polycomb-M33-deficient mice. *Development* 124**,** 721-729.

De Napoles, M., Mermoud, J.E., Wakao, R., Tang, Y.A., Endoh, M., Appanah, R., Nesterova, T.B., Silva, J., Otte, A.P., Vidal, M., Koseki, H., and Brockdorff, N. (2004). Polycomb group proteins Ring1A/B link ubiquitylation of histone H2A to heritable gene silencing and X inactivation. *Dev Cell* 7**,** 663-676.

Del Mar Lorente, M., Marcos-Gutierrez, C., Perez, C., Schoorlemmer, J., Ramirez, A., Magin, T., and Vidal, M. (2000). Loss- and gain-of-function mutations show a polycomb group function for Ring1A in mice. *Development* 127**,** 5093-5100.

Dey, B.K., Stalker, L., Schnerch, A., Bhatia, M., Taylor-Papidimitriou, J., and Wynder, C. (2008). The histone demethylase KDM5b/JARID1b plays a role in cell fate decisions by blocking terminal differentiation. *Mol Cell Biol* 28**,** 5312-5327.

Dodge, J.E., Kang, Y.K., Beppu, H., Lei, H., and Li, E. (2004). Histone H3-K9 methyltransferase ESET is essential for early development. *Mol Cell Biol* 24**,** 2478-2486.

Endoh, M., Endo, T.A., Endoh, T., Fujimura, Y., Ohara, O., Toyoda, T., Otte, A.P., Okano, M., Brockdorff, N., Vidal, M., and Koseki, H. (2008). Polycomb group proteins Ring1A/B are functionally linked to the core transcriptional regulatory circuitry to maintain ES cell identity. *Development* 135**,** 1513-1524.

Erhardt, S., Su, I.H., Schneider, R., Barton, S., Bannister, A.J., Perez-Burgos, L., Jenuwein, T., Kouzarides, T., Tarakhovsky, A., and Surani, M.A. (2003). Consequences of the depletion of zygotic and embryonic enhancer of zeste 2 during preimplantation mouse development. *Development* 130**,** 4235-4248.

Ernst, P., Mabon, M., Davidson, A.J., Zon, L.I., and Korsmeyer, S.J. (2004). An Mll-dependent Hox program drives hematopoietic progenitor expansion. *Curr Biol* 14**,** 2063-2069.

Faust, C., Schumacher, A., Holdener, B., and Magnuson, T. (1995). The eed mutation disrupts anterior mesoderm production in mice. *Development* 121**,** 273-285.

Forzati, F., Federico, A., Pallante, P., Fedele, M., and Fusco, A. (2012). Tumor suppressor activity of CBX7 in lung carcinogenesis. *Cell Cycle* 11**,** 1888-1891.

Gan, T., Jude, C.D., Zaffuto, K., and Ernst, P. (2010). Developmentally induced Mll1 loss reveals defects in postnatal haematopoiesis. *Leukemia* 24**,** 1732-1741.

Glaser, S., Lubitz, S., Loveland, K.L., Ohbo, K., Robb, L., Schwenk, F., Seibler, J., Roellig, D., Kranz, A., Anastassiadis, K., and Stewart, A.F. (2009). The histone 3 lysine 4 methyltransferase, Mll2, is only required briefly in development and spermatogenesis. *Epigenetics Chromatin* 2**,** 5.

Glaser, S., Schaft, J., Lubitz, S., Vintersten, K., Van Der Hoeven, F., Tufteland, K.R., Aasland, R., Anastassiadis, K., Ang, S.L., and Stewart, A.F. (2006). Multiple epigenetic maintenance factors implicated by the loss of Mll2 in mouse development. *Development* 133**,** 1423-1432.

Hu, G., Cui, K., Northrup, D., Liu, C., Wang, C., Tang, Q., Ge, K., Levens, D., Crane-Robinson, C., and Zhao, K. (2013). H2A.Z facilitates access of active and repressive complexes to chromatin in embryonic stem cell self-renewal and differentiation. *Cell Stem Cell* 12**,** 180-192.

Isono, K., Fujimura, Y., Shinga, J., Yamaki, M., J, O.W., Takihara, Y., Murahashi, Y., Takada, Y., Mizutani-Koseki, Y., and Koseki, H. (2005). Mammalian polyhomeotic homologues Phc2 and Phc1 act in synergy to mediate polycomb repression of Hox genes. *Mol Cell Biol* 25**,** 6694-6706.

Jiang, H., Shukla, A., Wang, X., Chen, W.Y., Bernstein, B.E., and Roeder, R.G. (2011). Role for Dpy-30 in ES cell-fate specification by regulation of H3K4 methylation within bivalent domains. *Cell* 144**,** 513-525.

Katoh-Fukui, Y., Tsuchiya, R., Shiroishi, T., Nakahara, Y., Hashimoto, N., Noguchi, K., and Higashinakagawa, T. (1998). Male-to-female sex reversal in M33 mutant mice. *Nature* 393**,** 688-692.

Kawazu, M., Saso, K., Tong, K.I., Mcquire, T., Goto, K., Son, D.O., Wakeham, A., Miyagishi, M., Mak, T.W., and Okada, H. (2011). Histone demethylase JMJD2B functions as a co-factor of estrogen receptor in breast cancer proliferation and mammary gland development. *PLoS One* 6**,** e17830.

Kuroki, S., Matoba, S., Akiyoshi, M., Matsumura, Y., Miyachi, H., Mise, N., Abe, K., Ogura, A., Wilhelm, D., Koopman, P., Nozaki, M., Kanai, Y., Shinkai, Y., and Tachibana, M. (2013). Epigenetic regulation of mouse sex determination by the histone demethylase Jmjd1a. *Science* 341**,** 1106-1109.

Lavial, F., Bessonnard, S., Ohnishi, Y., Tsumura, A., Chandrashekran, A., Fenwick, M.A., Tomaz, R.A., Hosokawa, H., Nakayama, T., Chambers, I., Hiiragi, T., Chazaud, C., and Azuara, V. (2012). Bmi1 facilitates primitive endoderm formation by stabilizing Gata6 during early mouse development. *Genes Dev* 26**,** 1445-1458.

Leeb, M., Pasini, D., Novatchkova, M., Jaritz, M., Helin, K., and Wutz, A. (2010). Polycomb complexes act redundantly to repress genomic repeats and genes. *Genes Dev* 24**,** 265-276.

Leeb, M., and Wutz, A. (2007). Ring1B is crucial for the regulation of developmental control genes and PRC1 proteins but not X inactivation in embryonic cells. *J Cell Biol* 178**,** 219-229.

Lin, W., Cao, J., Liu, J., Beshiri, M.L., Fujiwara, Y., Francis, J., Cherniack, A.D., Geisen, C., Blair, L.P., Zou, M.R., Shen, X., Kawamori, D., Liu, Z., Grisanzio, C., Watanabe, H., Minamishima, Y.A., Zhang, Q., Kulkarni, R.N., Signoretti, S., Rodig, S.J., Bronson, R.T., Orkin, S.H., Tuck, D.P., Benevolenskaya, E.V., Meyerson, M., Kaelin, W.G., Jr., and Yan, Q. (2011). Loss of the retinoblastoma binding protein 2 (RBP2) histone demethylase suppresses tumorigenesis in mice lacking Rb1 or Men1. *Proc Natl Acad Sci U S A* 108**,** 13379-13386.

Liu, Z., Zhou, S., Liao, L., Chen, X., Meistrich, M., and Xu, J. (2010). Jmjd1a demethylase-regulated histone modification is essential for cAMP-response element modulator-regulated gene expression and spermatogenesis. *J Biol Chem* 285**,** 2758-2770.

Loh, Y.H., Zhang, W., Chen, X., George, J., and Ng, H.H. (2007). Jmjd1a and Jmjd2c histone H3 Lys 9 demethylases regulate self-renewal in embryonic stem cells. *Genes Dev* 21**,** 2545-2557.

Lubitz, S., Glaser, S., Schaft, J., Stewart, A.F., and Anastassiadis, K. (2007). Increased apoptosis and skewed differentiation in mouse embryonic stem cells lacking the histone methyltransferase Mll2. *Mol Biol Cell* 18**,** 2356-2366.

Montgomery, N.D., Yee, D., Chen, A., Kalantry, S., Chamberlain, S.J., Otte, A.P., and Magnuson, T. (2005). The murine polycomb group protein Eed is required for global histone H3 lysine-27 methylation. *Curr Biol* 15**,** 942-947.

Morales Torres, C., Laugesen, A., and Helin, K. (2013). Utx is required for proper induction of ectoderm and mesoderm during differentiation of embryonic stem cells. *PLoS One* 8**,** e60020.

Morey, L., Pascual, G., Cozzuto, L., Roma, G., Wutz, A., Benitah, S.A., and Di Croce, L. (2012). Nonoverlapping functions of the Polycomb group Cbx family of proteins in embryonic stem cells. *Cell Stem Cell* 10**,** 47-62.

O'carroll, D., Erhardt, S., Pagani, M., Barton, S.C., Surani, M.A., and Jenuwein, T. (2001). The polycomb-group gene Ezh2 is required for early mouse development. *Mol Cell Biol* 21**,** 4330-4336.

O'loghlen, A., Munoz-Cabello, A.M., Gaspar-Maia, A., Wu, H.A., Banito, A., Kunowska, N., Racek, T., Pemberton, H.N., Beolchi, P., Lavial, F., Masui, O., Vermeulen, M., Carroll, T., Graumann, J., Heard, E., Dillon, N., Azuara, V., Snijders, A.P., Peters, G., Bernstein, E., and Gil, J. (2012). MicroRNA regulation of Cbx7 mediates a switch of Polycomb orthologs during ESC differentiation. *Cell Stem Cell* 10**,** 33-46.

Ohtani, K., Zhao, C., Dobreva, G., Manavski, Y., Kluge, B., Braun, T., Rieger, M.A., Zeiher, A.M., and Dimmeler, S. (2013). Jmjd3 controls mesodermal and cardiovascular differentiation of embryonic stem cells. *Circ Res* 113**,** 856-862.

Pasini, D., Bracken, A.P., Hansen, J.B., Capillo, M., and Helin, K. (2007). The polycomb group protein Suz12 is required for embryonic stem cell differentiation. *Mol Cell Biol* 27**,** 3769-3779.

Pasini, D., Bracken, A.P., and Helin, K. (2004). Polycomb group proteins in cell cycle progression and cancer. *Cell Cycle* 3**,** 396-400.

Pasini, D., Hansen, K.H., Christensen, J., Agger, K., Cloos, P.A., and Helin, K. (2008). Coordinated regulation of transcriptional repression by the RBP2 H3K4 demethylase and Polycomb-Repressive Complex 2. *Genes Dev* 22**,** 1345-1355.

Pedersen, M.T., Agger, K., Laugesen, A., Johansen, J.V., Cloos, P.A., Christensen, J., and Helin, K. (2014). The Demethylase JMJD2C Localizes to H3K4me3 Positive Transcription Start Sites and Is Dispensable for Embryonic Development. *Mol Cell Biol*.

Peters, A.H., O'carroll, D., Scherthan, H., Mechtler, K., Sauer, S., Schofer, C., Weipoltshammer, K., Pagani, M., Lachner, M., Kohlmaier, A., Opravil, S., Doyle, M., Sibilia, M., and Jenuwein, T. (2001). Loss of the Suv39h histone methyltransferases impairs mammalian heterochromatin and genome stability. *Cell* 107**,** 323-337.

Schmitz, S.U., Albert, M., Malatesta, M., Morey, L., Johansen, J.V., Bak, M., Tommerup, N., Abarrategui, I., and Helin, K. (2011). Jarid1b targets genes regulating development and is involved in neural differentiation. *EMBO J* 30**,** 4586-4600.

Schumacher, A., Faust, C., and Magnuson, T. (1996). Positional cloning of a global regulator of anterior-posterior patterning in mice. *Nature* 384**,** 648.

Shen, X., Liu, Y., Hsu, Y.J., Fujiwara, Y., Kim, J., Mao, X., Yuan, G.C., and Orkin, S.H. (2008). EZH1 mediates methylation on histone H3 lysine 27 and complements EZH2 in maintaining stem cell identity and executing pluripotency. *Mol Cell* 32**,** 491-502.

Shpargel, K.B., Sengoku, T., Yokoyama, S., and Magnuson, T. (2012). UTX and UTY demonstrate histone demethylase-independent function in mouse embryonic development. *PLoS Genet* 8**,** e1002964.

Stock, J.K., Giadrossi, S., Casanova, M., Brookes, E., Vidal, M., Koseki, H., Brockdorff, N., Fisher, A.G., and Pombo, A. (2007). Ring1-mediated ubiquitination of H2A restrains poised RNA polymerase II at bivalent genes in mouse ES cells. *Nat Cell Biol* 9**,** 1428-1435.

Stoller, J.Z., Huang, L., Tan, C.C., Huang, F., Zhou, D.D., Yang, J., Gelb, B.D., and Epstein, J.A. (2010). Ash2l interacts with Tbx1 and is required during early embryogenesis. *Exp Biol Med (Maywood)* 235**,** 569-576.

Suzuki, M., Mizutani-Koseki, Y., Fujimura, Y., Miyagishima, H., Kaneko, T., Takada, Y., Akasaka, T., Tanzawa, H., Takihara, Y., Nakano, M., Masumoto, H., Vidal, M., Isono, K., and Koseki, H. (2002). Involvement of the Polycomb-group gene Ring1B in the specification of the anterior-posterior axis in mice. *Development* 129**,** 4171-4183.

Tachibana, M., Sugimoto, K., Nozaki, M., Ueda, J., Ohta, T., Ohki, M., Fukuda, M., Takeda, N., Niida, H., Kato, H., and Shinkai, Y. (2002). G9a histone methyltransferase plays a dominant role in euchromatic histone H3 lysine 9 methylation and is essential for early embryogenesis. *Genes Dev* 16**,** 1779-1791.

Tachibana, M., Ueda, J., Fukuda, M., Takeda, N., Ohta, T., Iwanari, H., Sakihama, T., Kodama, T., Hamakubo, T., and Shinkai, Y. (2005). Histone methyltransferases G9a and GLP form heteromeric complexes and are both crucial for methylation of euchromatin at H3-K9. *Genes Dev* 19**,** 815-826.

Takihara, Y., Tomotsune, D., Shirai, M., Katoh-Fukui, Y., Nishii, K., Motaleb, M.A., Nomura, M., Tsuchiya, R., Fujita, Y., Shibata, Y., Higashinakagawa, T., and Shimada, K. (1997). Targeted disruption of the mouse homologue of the Drosophila polyhomeotic gene leads to altered anteroposterior patterning and neural crest defects. *Development* 124**,** 3673-3682.

Terranova, R., Agherbi, H., Boned, A., Meresse, S., and Djabali, M. (2006). Histone and DNA methylation defects at Hox genes in mice expressing a SET domain-truncated form of Mll. *Proc Natl Acad Sci U S A* 103**,** 6629-6634.

Van Der Lugt, N.M., Domen, J., Linders, K., Van Roon, M., Robanus-Maandag, E., Te Riele, H., Van Der Valk, M., Deschamps, J., Sofroniew, M., Van Lohuizen, M., and Et Al. (1994). Posterior transformation, neurological abnormalities, and severe hematopoietic defects in mice with a targeted deletion of the bmi-1 proto-oncogene. *Genes Dev* 8**,** 757-769.

Van Der Stoop, P., Boutsma, E.A., Hulsman, D., Noback, S., Heimerikx, M., Kerkhoven, R.M., Voncken, J.W., Wessels, L.F., and Van Lohuizen, M. (2008). Ubiquitin E3 ligase Ring1b/Rnf2 of polycomb repressive complex 1 contributes to stable maintenance of mouse embryonic stem cells. *PLoS One* 3**,** e2235.

Villasante, A., Piazzolla, D., Li, H., Gomez-Lopez, G., Djabali, M., and Serrano, M. (2011). Epigenetic regulation of Nanog expression by Ezh2 in pluripotent stem cells. *Cell Cycle* 10**,** 1488-1498.

Voncken, J.W., Roelen, B.A., Roefs, M., De Vries, S., Verhoeven, E., Marino, S., Deschamps, J., and Van Lohuizen, M. (2003). Rnf2 (Ring1b) deficiency causes gastrulation arrest and cell cycle inhibition. *Proc Natl Acad Sci U S A* 100**,** 2468-2473.

Wan, M., Liang, J., Xiong, Y., Shi, F., Zhang, Y., Lu, W., He, Q., Yang, D., Chen, R., Liu, D., Barton, M., and Songyang, Z. (2013). The trithorax group protein Ash2l is essential for pluripotency and maintaining open chromatin in embryonic stem cells. *J Biol Chem* 288**,** 5039-5048.

Wang, C., Lee, J.E., Cho, Y.W., Xiao, Y., Jin, Q., Liu, C., and Ge, K. (2012). UTX regulates mesoderm differentiation of embryonic stem cells independent of H3K27 demethylase activity. *Proc Natl Acad Sci U S A* 109**,** 15324-15329.

Wang, J., Scully, K., Zhu, X., Cai, L., Zhang, J., Prefontaine, G.G., Krones, A., Ohgi, K.A., Zhu, P., Garcia-Bassets, I., Liu, F., Taylor, H., Lozach, J., Jayes, F.L., Korach, K.S., Glass, C.K., Fu, X.D., and Rosenfeld, M.G. (2007). Opposing LSD1 complexes function in developmental gene activation and repression programmes. *Nature* 446**,** 882-887.

Wang, Y., Zhang, H., Chen, Y., Sun, Y., Yang, F., Yu, W., Liang, J., Sun, L., Yang, X., Shi, L., Li, R., Li, Y., Zhang, Y., Li, Q., Yi, X., and Shang, Y. (2009). LSD1 is a subunit of the NuRD complex and targets the metastasis programs in breast cancer. *Cell* 138**,** 660-672.

Welstead, G.G., Creyghton, M.P., Bilodeau, S., Cheng, A.W., Markoulaki, S., Young, R.A., and Jaenisch, R. (2012). X-linked H3K27me3 demethylase Utx is required for embryonic development in a sex-specific manner. *Proc Natl Acad Sci U S A* 109**,** 13004-13009.

Wysocka, J., Swigut, T., Milne, T.A., Dou, Y., Zhang, X., Burlingame, A.L., Roeder, R.G., Brivanlou, A.H., and Allis, C.D. (2005). WDR5 associates with histone H3 methylated at K4 and is essential for H3 K4 methylation and vertebrate development. *Cell* 121**,** 859-872.

Yagi, H., Deguchi, K., Aono, A., Tani, Y., Kishimoto, T., and Komori, T. (1998). Growth disturbance in fetal liver hematopoiesis of Mll-mutant mice. *Blood* 92**,** 108-117.

Yeap, L.S., Hayashi, K., and Surani, M.A. (2009). ERG-associated protein with SET domain (ESET)-Oct4 interaction regulates pluripotency and represses the trophectoderm lineage. *Epigenetics Chromatin* 2**,** 12.

Yin, F., Lan, R., Zhang, X., Zhu, L., Chen, F., Xu, Z., Liu, Y., Ye, T., Sun, H., Lu, F., and Zhang, H. (2014). LSD1 Regulates Pluripotency of Embryonic Stem/Carcinoma Cells through Histone Deacetylase 1-Mediated Deacetylation of Histone H4 at Lysine 16. *Mol Cell Biol* 34**,** 158-179.

Yu, B.D., Hess, J.L., Horning, S.E., Brown, G.A., and Korsmeyer, S.J. (1995). Altered Hox expression and segmental identity in Mll-mutant mice. *Nature* 378**,** 505-508.

Yuan, P., Han, J., Guo, G., Orlov, Y.L., Huss, M., Loh, Y.H., Yaw, L.P., Robson, P., Lim, B., and Ng, H.H. (2009). Eset partners with Oct4 to restrict extraembryonic trophoblast lineage potential in embryonic stem cells. *Genes Dev* 23**,** 2507-2520.
